# Lipid-derived microbial metabolites at the gut-liver interface: regulation of intestinal barrier function, immune signaling, and metabolic inflammation

**DOI:** 10.3389/fphys.2026.1881697

**Published:** 2026-07-15

**Authors:** Carlo Acierno, Alfredo Caturano, Fannia Barletta, Luca Rinaldi, Ferdinando Carlo Sasso, Luigi Elio Adinolfi, Riccardo Nevola

**Affiliations:** 1 Department of Internal Medicine, San Carlo Hospital, Potenza, Italy; 2 Department of Medicine and Surgery, University of Basilicata, Potenza, Italy; 3 Department of Human Sciences and Promotion of the Quality of Life, San Raffaele Roma Open University, Rome, Italy; 4 Department of Anesthesiology and Intensive Care, San Carlo Hospital, Potenza, Italy; 5 Department of Medicine and Health Science “Vincenzo Tiberio”, Università degli Studi del Molise, Campobasso, Italy; 6 Department of Advanced Medical and Surgical Sciences, University of Campania “Luigi Vanvitelli”, Napoli, Italy; 7 Liver Unit, “A. Landolfi” Hospital, AORN San Giuseppe Moscati, Solofra, Italy

**Keywords:** bacterial sphingolipids, bile acids, gut-liver axis, intestinal barrier, MASLD, PUFA-derived mediators, short-chain fatty acids, Trimethylamine N-oxide

## Abstract

Lipid-derived and lipid-related microbial metabolites form a functional interface between the gut microbiota, intestinal barrier integrity, mucosal immunity, hepatic metabolism, and systemic immunometabolic homeostasis. At the gut-liver axis, these molecules should not be regarded as passive end-products of microbial metabolism, but as compartment-sensitive signals whose biological effects depend on site of production, mucosal exposure, epithelial utilization, portal delivery, hepatic handling, receptor distribution, and inflammatory context. This narrative review examines five major metabolite classes: short-chain fatty acids, including branched-chain fatty acids; microbially transformed bile acids; bacterial sphingolipids; PUFA-derived or microbially modulated lipid mediators; and choline-, carnitine-, and phospholipid-derived co-metabolites such as trimethylamine N-oxide. We discuss how these pathways regulate tight-junction architecture, epithelial energy metabolism, mucus-associated defense, antimicrobial programs, innate and adaptive immune-cell signaling, bile-acid receptor networks, and portal inflammatory exposure. The evidence is most mature for classical short-chain fatty acids and bile-acid signaling, emerging but mechanistically compelling for bacterial sphingolipids and PUFA-derived mediators, context-dependent for TMAO, and still limited for aging-specific and sarcopenia-related extrapolations. Across classes, translation is constrained by incomplete causal inference, preclinical predominance, heterogeneous human evidence, inconsistent metabolomic and lipidomic standardization, and the non-equivalence of fecal concentrations, mucosal exposure, portal delivery, and systemic availability. Future studies should move beyond single-metabolite associations toward longitudinal, multi-compartment, mechanism-informed models integrating targeted metabolomics/lipidomics, permeability markers, immune phenotyping, liver and cardiometabolic endpoints, and functional outcomes relevant to frailty and sarcopenia.

## 1 Introduction: the gut-liver axis as an immunometabolic interface

The gut-liver axis is a bidirectional immunometabolic circuit in which portal blood flow, epithelial barrier integrity, bile-acid metabolism, innate immune sensing, and microbial ecology converge. The liver is continuously exposed to nutrients, microbial products, and intestinal metabolites delivered through the portal vein. Even moderate changes in intestinal permeability or microbial metabolic output can reshape the antigenic and metabolic landscape encountered by Kupffer cells, hepatocytes, hepatic stellate cells, and sinusoidal endothelial cells (Albillos et al., 2020; Tilg et al., 2022; Hsu and Schnabl, 2023; Pabst et al., 2023; Acierno et al., 2025d).

The intestinal barrier is not a passive anatomical wall. It integrates epithelial cells, tight junctions, adherens junctions, mucus, antimicrobial peptides, secretory immunoglobulins, resident myeloid cells, lymphocytes, and metabolite-sensitive signaling pathways. Its principal function is selective compartmentalization – allowing nutrient uptake and commensal tolerance while preventing uncontrolled translocation of microbial patterns and inflammatory cues (Turner, 2009; Bischoff et al., 2014; Peterson and Artis, 2014; Camilleri, 2019).

Lipid-derived microbial metabolites offer a mechanistically coherent framework for interpreting this interface. Short-chain fatty acids (SCFAs), microbially transformed bile acids, bacterial sphingolipids, polyunsaturated fatty-acid-derived mediators, and choline/carnitine/phospholipid-derived products can function as receptor ligands, energetic substrates, epigenetic modulators, redox signals, or immunoregulatory mediators. Through these roles, they connect diet, microbial ecology, epithelial resilience, immune-cell programming, and systemic metabolism (Koh et al., 2016; Rooks and Garrett, 2016; Blacher et al., 2017; Husted et al., 2017; Gasaly et al., 2021).

This framework is clinically relevant above all in metabolic liver disease. MASLD, obesity, type 2 diabetes, and cardiometabolic disease share overlapping disturbances in insulin signaling, lipid handling, bile-acid homeostasis, adipose-tissue inflammation, intestinal permeability, and low-grade systemic inflammation -- making the gut-liver axis a plausible model for understanding how microbial lipid metabolism contributes to the transition from metabolic adaptation to chronic inflammatory injury (Leung et al., 2016; Aron-Wisnewsky et al., 2020).

Previous reviews have extensively addressed the gut microbiota in liver disease, SCFA biology, bile-acid signaling, and the broader concept of microbial dysbiosis in MASLD. However, several gaps remain insufficiently integrated. First, lipid-derived and lipid-related microbial metabolites are often discussed as separate biochemical classes rather than as compartment-dependent signals acting across the intestinal lumen, mucus layer, epithelium, portal circulation, liver, and systemic metabolic tissues. Second, fecal metabolite abundance is frequently interpreted as a proxy for biological activity, although production, epithelial utilization, mucosal exposure, portal delivery, hepatic processing, and systemic receptor engagement are not interchangeable. Third, emerging lipid classes such as bacterial sphingolipids, PUFA-derived mediators, oxylipins, and endocannabinoid-like lipids are less mature than SCFAs and bile acids, yet they are increasingly relevant to immunometabolic interpretation. Finally, the translational literature remains uneven, with robust mechanistic evidence for some pathways and more limited human validation for others.

**Figure 1:**
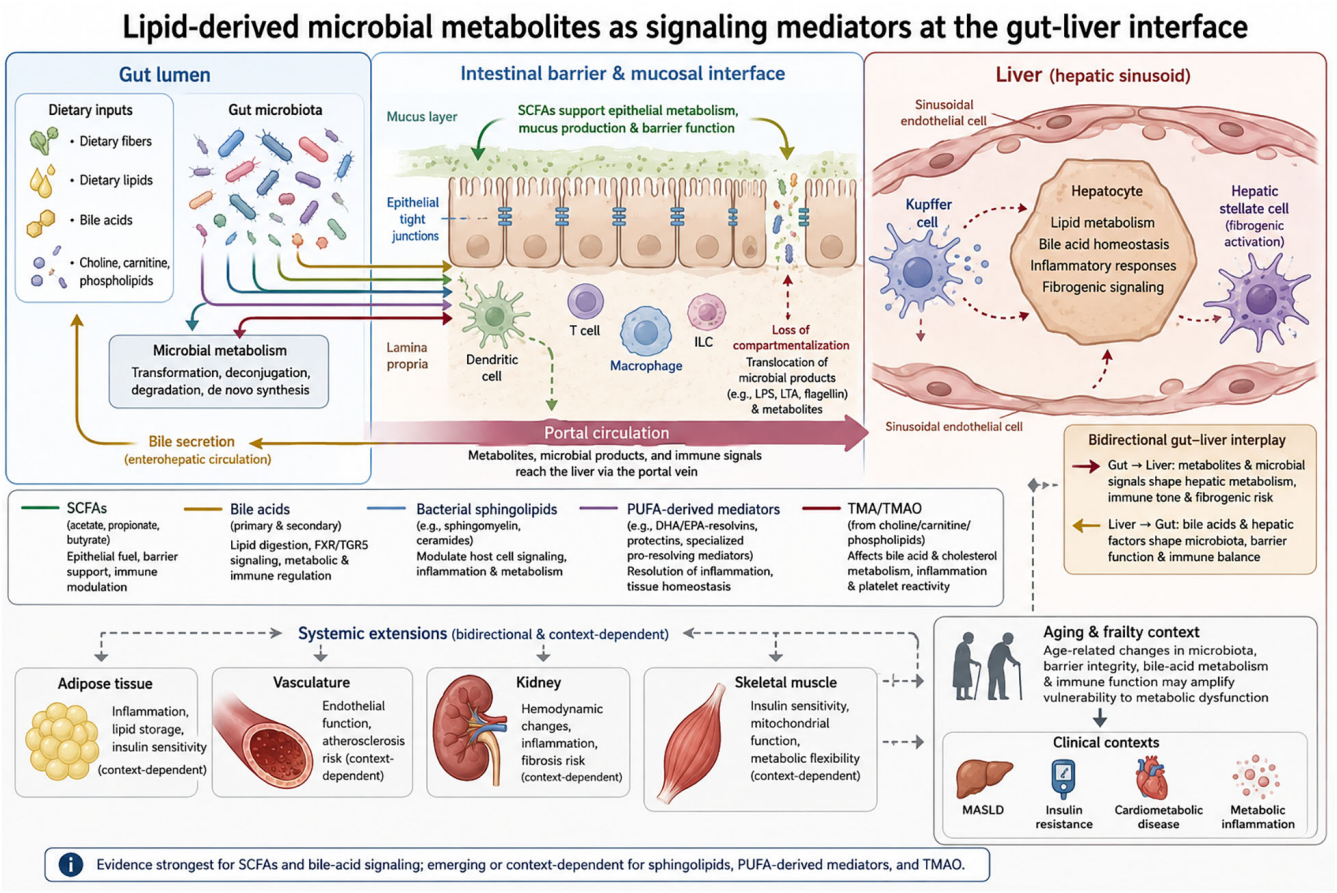
Lipid-derived microbial metabolites at the gut-liver immunometabolic interface. Conceptual framework showing how microbial and diet-host-derived lipid signals link gut microbiota ecology to intestinal barrier function, mucus and tight-junction integrity, immune signaling, portal delivery of gut-derived cues, hepatic immunometabolic stress, MASLD-related metabolic inflammation, and systemic cardiometabolic or aging-associated phenotypes. The arrows indicate biologically plausible and literature-supported connections discussed in the review, and should not be interpreted as a single linear or universally causal sequence.

This review is therefore organized around a compartment-aware translational framework. We first define the relevant classes of lipid-derived or lipid-related microbial metabolites, then examine how they influence epithelial barrier biology, mucus-associated defense, immune-cell programming, bile-acid receptor signaling, and hepatic immunometabolic stress. We then extend the model to MASLD, insulin resistance, cardiometabolic disease, skeletal muscle vulnerability, and aging-related frailty. The aim is not to propose a single-metabolite explanation for metabolic disease, but to critically distinguish established mechanisms from plausible, incompletely validated translational hypotheses and to identify methodological requirements for future gut-liver metabolomics studies. Taken together, these elements support a compartment-aware view in which microbial lipid metabolism, barrier integrity, portal transport, hepatic sensing, and systemic immunometabolic outcomes form a connected network rather than isolated pathways (**Figure 1**).

## 2 Scope and narrative approach

This manuscript is a narrative review. It does not follow a systematic-review methodology, PRISMA workflow, independent screening process, or formal risk-of-bias assessment. The synthesis is organized around biological plausibility, consistency across mechanistic and translational evidence, and relevance to gut-liver immunometabolism.

A targeted narrative search was performed to support the conceptual synthesis. PubMed/MEDLINE, Scopus, Web of Science, and Google Scholar were used as primary sources, prioritizing peer-reviewed articles published from 2000 to 2026 and landmark earlier studies when mechanistically necessary. Search terms spanned the following domains: “gut-liver axis”, “microbial metabolites”, “short-chain fatty acids”, “bile acids”, “microbial sphingolipids”, “oxylipins”, “polyunsaturated fatty acids”, “trimethylamine N-oxide”, “intestinal barrier”, “metabolic inflammation”, “MASLD”, “NAFLD”, “insulin resistance”, “cardiometabolic disease”, “aging”, “inflammaging”, and “frailty”. Eligible sources included mechanistic experimental studies, translational human studies, clinical cohorts, consensus documents, and authoritative reviews on microbial lipid metabolism, epithelial barrier biology, immune signaling, liver disease, and cardiometabolic outcomes. Studies were excluded when unrelated to microbial or lipid-derived metabolism, focused solely on taxonomic dysbiosis without functional interpretation, or addressed claims outside the gut-liver immunometabolic interface.

The review covers metabolites directly produced by gut microbes and metabolites that are host- or diet-derived but substantially transformed by microbial activity. SCFAs arise primarily from bacterial fermentation; secondary bile acids from microbial transformation of host-produced bile acids; and TMAO from microbial trimethylamine generation followed by hepatic oxidation. The manuscript therefore uses the term “lipid-derived or lipid-related microbial metabolites” where biochemical origin is shared between diet, host, and microbiota (Wahlstrom et al., 2016; Tang and Hazen, 2017; Rowland et al., 2018).

The level of certainty is intentionally differentiated throughout. Stronger wording is reserved for pathways supported by convergent mechanistic and translational data — particularly SCFAs, epithelial metabolism, and bile-acid receptor signaling. More cautious language is used for bacterial sphingolipids, oxylipin-like mediators, endocannabinoid-like pathways, and aging-specific extrapolations, where evidence remains biologically plausible but clinically less mature. The narrative also favors mechanisms that map onto clinically measurable domains: permeability, bile-acid pool composition, targeted lipid metabolomics, inflammatory tone, metabolic phenotype, and liver disease stage.

## 3 Major classes of lipid-derived and lipid-related microbial metabolites

### 3.1 Short-chain fatty acids and microbial fatty-acid fermentation products

Acetate, propionate, and butyrate are the classical, best-characterized microbial metabolites connecting dietary carbohydrate fermentation to host physiology (**Table 1**). Derived from bacterial processing of non-digestible polysaccharides and related fermentable substrates, they function as energetic substrates, ligands for G-protein-coupled receptors -- including FFAR3/GPR41, FFAR2/GPR43, and GPR109A -- and epigenetic regulators through histone deacetylase inhibition. Butyrate is especially relevant to colonic epithelial biology because it supports oxidative metabolism in colonocytes and contributes to epithelial barrier integrity (Hamer et al., 2008; Donohoe et al., 2011; Morrison and Preston, 2016; Makki et al., 2018; Mann et al., 2024).

**Table 1:** Major classes of lipid-derived or lipid-related microbial metabolites relevant to the gut-liver axis.

Metabolite class	Functional origin	Dominant host targets/signaling axes	Translational interpretation	Key references
Classical short-chain fatty acids (SCFAs)	Bacterial fermentation of non-digestible carbohydrates; acetate, propionate, and butyrate as principal products.	Colonocyte oxidative metabolism; FFAR2/FFAR3; GPR109A; HDAC inhibition; Treg induction; macrophage programming; epithelial AMPK and HIF-related barrier pathways.	Most mature class for epithelial and immune homeostasis; effects are concentration-, receptor-, compartment-, and disease-context dependent. Fecal concentrations should not be interpreted as direct proxies for mucosal exposure, portal delivery, or systemic receptor engagement.	(Hamer et al., 2008; Maslowski et al., 2009; Donohoe et al., 2011; Arpaia et al., 2013; Furusawa et al., 2013; Smith et al., 2013; Macia et al., 2015; Koh et al., 2016; Morrison and Preston, 2016; Boets et al., 2017; Makki et al., 2018; Müller et al., 2019; Sakata, 2019; Kim, 2021; Mann et al., 2024)
Branched-chain SCFAs (BCFAs)	Microbial fermentation of branched-chain amino acids; isobutyrate mainly from valine, isovalerate from leucine, and 2-methylbutyrate from isoleucine.	Protein-fermentation readouts; BCAA-related microbial metabolism; possible effects on adipocyte metabolism, glucose handling, epithelial stress, and inflammatory tone.	Emerging and context-dependent. BCFAs should be distinguished from acetate, propionate, and butyrate. Causal links with insulin resistance or barrier dysfunction remain incompletely established; circulating and fecal BCFA pools provide different biological information.	(Heimann et al., 2016; Rios-Covian et al., 2020; Aslamy et al., 2024)
Microbially transformed bile acids	Host-derived primary bile acids modified by microbial deconjugation, dehydroxylation, oxidation/epimerization, and related transformations.	Ileal FXR-FGF19/FGF15 feedback; colonic FXR/TGR5 context; PXR-linked pathways; hepatointestinal feedback; epithelial defense; glucose/lipid metabolism; Treg/Th17 balance.	Strong gut-liver signaling class. Biological effects depend on bile-acid pool composition, hydrophobicity, conjugation state, receptor affinity, ileal versus colonic localization, and microbial transformation.	(Makishima et al., 1999; Parks et al., 1999; Kawamata et al., 2003; Ridlon et al., 2006; Thomas et al., 2009; Sayin et al., 2013; Li and Chiang, 2014; Wahlstrom et al., 2016; Hang et al., 2019; Campbell et al., 2020; Chiang and Ferrell, 2020; Song et al., 2020; Fiorucci et al., 2021; Paik et al., 2022; Collins et al., 2023)
Bacterial sphingolipids	Direct lipid production by selected intestinal taxa, particularly *Bacteroides* species; biological effects vary according to commensal, pathobiont, or inflammatory context.	Bacterial fitness and symbiosis; NKT-cell homeostasis via CD1d-restricted lipid antigen presentation; epithelial-immune crosstalk; host ceramide pool; sphingolipid-related immunometabolic signaling.	Mechanistically compelling but clinically less mature than SCFAs and bile acids. Commensal *Bacteroides*-derived sphingolipids may support homeostasis; altered ceramide signaling may interact with insulin resistance and inflammation. Direct human evidence in MASLD remains limited.	(Heung et al., 2006; An et al., 2011; Holland et al., 2011; An et al., 2014; Heaver et al., 2018; Brown et al., 2019; Johnson et al., 2020; Le et al., 2022; Bai et al., 2023; Overbeek et al., 2024)
PUFA-derived microbial or microbially modulated mediators	Microbial transformation of dietary or host-derived PUFAs into hydroxy-fatty acids, conjugated linoleic-acid intermediates, oxylipin-like mediators, and lipid networks linked to the endocannabinoidome.	GPR40/MEK/ERK signaling; PPAR-linked lipid networks; epithelial barrier modulation; adipogenesis; endocannabinoidome-related inflammatory and metabolic signaling.	Emerging field. Some mediators are directly microbial products; others are microbially modulated host-diet lipid networks. Human translational evidence remains incomplete and should be separated from experimental mechanistic data.	(Devillard et al., 2007; Muccioli et al., 2010; Kishino et al., 2013; Miyamoto et al., 2015; Lacroix et al., 2019; Miyamoto et al., 2019; Castonguay-Paradis et al., 2020; Castonguay-Paradis et al., 2023; Roussel et al., 2024; Roussel et al., 2025)
Choline-, carnitine-, and phospholipid-derived metabolites (TMA/TMAO)	Microbial trimethylamine (TMA) generation from dietary precursors followed by hepatic FMO3-dependent oxidation to TMAO; diet-microbe-liver co-metabolic sequence.	Hepatic FMO3 activity; bile-acid and cholesterol metabolism; endothelial and vascular inflammatory pathways; renal-host interactions; hepatic inflammatory/fibrotic signaling in experimental models.	Useful systemic co-metabolic paradigm; relevant to the gut-liver axis through microbial TMA production and hepatic oxidation. Interpretation confounded by diet, renal function, hepatic oxidation capacity, microbiome ecology, and comorbidity; should not be treated as a stand-alone causal mediator.	(Wang et al., 2011; Koeth et al., 2013; Tang et al., 2013; Miao et al., 2015; Warrier et al., 2015; Tang and Hazen, 2017; Tan et al., 2019; He et al., 2020; Flores‐Guerrero et al., 2021; León-Mimila et al., 2021; Theofilis et al., 2022, 200, 201)

Branched-chain short-chain fatty acids (BCFAs) -- principally isobutyrate, isovalerate, and 2-methylbutyrate -- constitute a distinct subgroup produced mainly from bacterial catabolism of proteins and branched-chain amino acids rather than from carbohydrate fermentation. Fecal BCFA abundance varies with age, BMI, dietary patterns, and anthropometric factors in human cohorts (Rios-Covian et al., 2020), and may shift together with changes in microbial community structure. Their metabolic interpretation should not be conflated with that of acetate, propionate, or butyrate. *In vitro* evidence indicates that isobutyrate and isovalerate can modulate lipolysis, glucose uptake, and lipogenesis in adipocytes (Heimann et al., 2016), supporting biological activity outside the intestinal lumen; however, these findings derive from isolated adipocyte models and cannot be directly extrapolated to intestinal or hepatic physiology. Importantly, human plasma BCFA data suggest that associations with metabolic health are compartment-dependent: in the MILES cohort, higher circulating branched SCFAs were associated with more favorable glucose homeostasis (Aslamy et al., 2024). BCFAs should therefore be included as protein- and BCAA-derived microbial fermentation products with emerging metabolic relevance, but they should not be framed as uniformly adverse markers of insulin resistance or as functional analogs of classical SCFAs.

SCFAs also shape mucosal immunity. Butyrate and, to varying degrees, other classical SCFAs have been linked in preclinical studies to colonic regulatory T-cell differentiation, peripheral Treg generation, macrophage modulation, neutrophil responses, and inflammasome regulation (Maslowski et al., 2009; Arpaia et al., 2013; Furusawa et al., 2013; Smith et al., 2013; Macia et al., 2015; Kim, 2021). These findings support a role for gut-derived SCFAs as modulators of barrier-associated immune tone. However, most mechanistic data originate from animal models or cell culture, and immune effects vary substantially with concentration, receptor expression, epithelial versus immune-cell context, microbial community composition, disease stage, and timing. SCFAs are therefore best described as context-sensitive immunometabolic signals that are neither unconditionally protective nor uniformly pro-inflammatory (Kim et al., 2013; Parada Venegas et al., 2019; Ney et al., 2023).

A critical methodological issue concerns the interpretation of SCFA measurements across biological compartments. Fecal SCFA concentration reflects net luminal excretion -- a composite of microbial production, colonocytic utilization, passive and active absorption, colonic transit, and microbial ecology -- and should not be treated as a direct index of any individual process. Human stable-isotope tracing demonstrates that a substantial proportion of colonically derived SCFAs is absorbed and metabolized before reaching systemic circulation (Boets et al., 2017; Sakata, 2019), such that fecal output captures residual excretion rather than total production or systemic availability. Conversely, in a human observational cohort, higher fecal SCFA excretion was associated with dysbiosis, obesity, hypertension, cardiometabolic risk factors, and a marker of gut permeability (De La Cuesta-Zuluaga et al., 2018) rather than with a uniformly favorable phenotype. When circulating rather than fecal SCFAs were measured in a human metabolic study, they were more closely linked to GLP-1 concentrations, lipolysis, and insulin sensitivity (Müller et al., 2019). Fecal, mucosal, portal, and systemic SCFA pools are therefore not interchangeable: each captures a different biological compartment, and inferring gut-liver signaling from fecal abundance alone introduces substantial interpretive uncertainty. Technical approaches to address this limitation through multi-compartment sampling, stable-isotope tracing, and functional readouts are discussed in Section 9.

For the gut-liver axis, classical SCFAs and BCFAs should be interpreted as context-dependent microbial fermentation products whose relevance to MASLD depends on epithelial compartmentalization, mucosal utilization, portal delivery, hepatic sensing, insulin resistance, and inflammatory tone. SCFAs may contribute to reduced portal inflammatory burden when barrier integrity and epithelial metabolism are preserved, whereas altered fermentation patterns, impaired compartmentalization, and metabolic inflammation may change their biological interpretation. This framework is especially pertinent in metabolic liver disease, where barrier dysfunction, microbial translocation, adipose inflammation, and insulin resistance can reinforce one another (Cani et al., 2007; Cani et al., 2008; Vinolo et al., 2011; Trompette et al., 2014; Schroeder and Backhed, 2016; De Munck et al., 2020). However, the directionality and magnitude of SCFA and BCFA contributions to these interactions remain incompletely defined in human MASLD and should be treated as a mechanistically plausible framework rather than an established causal chain.

### 3.2 Microbially transformed bile acids

Bile acids are a central class at the gut-liver interface because their biology is intrinsically enterohepatic. The liver synthesizes primary bile acids, which are secreted into bile and released into the intestine, where microbial deconjugation, dehydroxylation, and related transformations generate a receptor-diverse bile-acid pool (Ridlon et al., 2006; Li and Chiang, 2014; Wahlstrom et al., 2016; Collins et al., 2023).

The biological reach of bile acids extends far beyond lipid absorption. FXR and TGR5 translate bile-acid pool composition into programs that regulate bile-acid synthesis and transport, glucose and lipid metabolism, epithelial defense, intestinal permeability, macrophage function, and systemic metabolic tone (Makishima et al., 1999; Parks et al., 1999; Kawamata et al., 2003; Thomas et al., 2009; Chiang and Ferrell, 2020; Fiorucci et al., 2021). Microbial bile-acid metabolites also influence adaptive immunity — particularly the Treg/Th17 axis — making bile acids a strong mechanistic bridge between microbial lipid metabolism and mucosal immune programming (Hang et al., 2019; Campbell et al., 2020; Song et al., 2020; Paik et al., 2022).

The bile-acid pool is better understood as a dynamic signaling code than a uniform class of detergents. Hydrophobicity, conjugation state, microbial transformation, receptor affinity, ileal versus colonic localization, and enterohepatic cycling together determine whether bile acids reinforce barrier defense, support metabolic homeostasis, or contribute to epithelial stress (Taylor and Green, 2018; Fiorucci et al., 2021; Visekruna and Luu, 2021).

### 3.3 Bacterial sphingolipids and host lipid signaling

Bacterial sphingolipids are an emerging class of microbiota-derived lipid signals produced by a restricted set of intestinal taxa, most characteristically *Bacteroides* species (An et al., 2011; Heaver et al., 2018; Bai et al., 2023). They differ from SCFAs and bile acids less in biological plausibility than in evidentiary maturity: whereas SCFA and bile-acid signaling rest on defined host receptor pathways and a broader human translational literature, bacterial sphingolipid biology is still anchored largely in microbial genetics, structural lipidomics, gnotobiotic models, and pathway-level host-microbe studies. This section therefore treats *Bacteroides*-derived sphingolipids as concrete mechanistic examples while keeping translational claims deliberately measured.

The best-characterized example concerns intestinal immune homeostasis. In gnotobiotic models, sphingolipids from symbiotic *Bacteroides* can shape the homeostasis of intestinal invariant natural killer T cells through CD1d-restricted lipid antigen presentation rather than through a nonspecific immunostimulatory effect (An et al., 2014). Consistent with a structural role in host-microbe accommodation, *Bacteroides* strains deficient in sphingolipid synthesis show altered colonization fitness and disturbed intestinal homeostasis, with changes in mucosal inflammatory tone (An et al., 2011; Brown et al., 2019). These data support a more precise statement than the generic claim that bacterial sphingolipids “regulate NKT cells”: symbiotic *Bacteroides*-derived sphingolipids can modulate intestinal iNKT-cell homeostasis through CD1d-associated lipid antigen biology.

A second, partly distinct property is metabolic. Gut bacterial sphingolipids can enter host metabolic pathways, and isotope-resolved analyses in gnotobiotic systems indicate that microbially produced sphingolipids and their downstream metabolites can be incorporated into host lipid pools and affect the host ceramide pool (Johnson et al., 2020). Loss of *Bacteroides* sphingolipid synthesis also reshapes host sphingolipid and ceramide metabolism (Brown et al., 2019). Because host ceramide accumulation is mechanistically linked to lipotoxic inflammation and insulin resistance (Holland et al., 2011), these findings raise a biologically plausible connection between microbial sphingolipid input and host immunometabolic signaling. This connection should remain evidence-graded: host ceramide biology supports mechanistic plausibility, but it does not prove that bacterial sphingolipids cause insulin resistance, MASLD progression, or cardiometabolic disease in humans.

The biological consequences of bacterial sphingolipids are context-dependent rather than uniformly beneficial or harmful. In a commensal or symbiotic context, *Bacteroides*-derived sphingolipids appear to support host-microbe accommodation and intestinal homeostasis (An et al., 2014; Brown et al., 2019). In pathogenic or pathobiont contexts, microbial sphingolipid pathways may instead contribute to microbial fitness, immune interaction, or virulence-associated phenotypes (Heung et al., 2006). A simple protective-versus-pathogenic dichotomy is therefore not supported. The same broad chemical class can exert divergent effects depending on the producing organism, lipid structure, anatomical compartment, host inflammatory state, and metabolic background (Heaver et al., 2018; Bai et al., 2023).

A gut-liver extension is emerging but remains predominantly preclinical. In a mouse model of hepatic steatosis, manipulation of gut bacterial sphingolipid synthesis altered hepatic lipid accumulation, and bacterial sphingolipid was traced from the intestine to the liver, supporting the concept that microbial sphingolipid output can reach hepatic metabolic pathways (Le et al., 2022). Human evidence remains indirect. A HELIUS case-cohort analysis linked gut microbiome features and plasma sphingolipid patterns, including ceramide-dominant components, with incident type 2 diabetes, but it did not establish bacterial sphingolipids as causal mediators and did not specifically address MASLD (Overbeek et al., 2024). Taken together, *Bacteroides*-derived sphingolipids are mechanistically credible modulators of intestinal immune and metabolic biology with a plausible preclinical gut-liver dimension, yet direct human evidence connecting bacterial sphingolipids to MASLD progression, frailty, or cardiometabolic endpoints remains insufficient to support firm translational claims.

### 3.4 PUFA-derived mediators, hydroxy-fatty acids, oxylipins, and endocannabinoid-like lipids

The microbiota can transform dietary polyunsaturated fatty acids (PUFAs) into bioactive derivatives, and these transformations can influence host lipid composition and metabolic phenotype in experimental models. 10-Hydroxy-cis-12-octadecenoic acid (10-HYA) is the most thoroughly characterized example, but it represents only one node of a broader microbial PUFA-metabolizing repertoire and should not be taken as representative of the whole field (Kishino et al., 2013; Miyamoto et al., 2015; Miyamoto et al., 2019).

Human gut bacteria metabolize linoleic acid through more than one enzymatic route. Lactic acid bacteria can hydrate, oxidize, isomerize, and saturate unsaturated fatty acids, generating a spectrum of intermediates that includes hydroxy-fatty acids, oxo/keto-fatty acid intermediates, and partially saturated species (Kishino et al., 2013). Distinct human intestinal isolates can also convert linoleic acid into conjugated linoleic-acid-related intermediates through separate pathways, indicating that microbial PUFA metabolism is taxonomically and biochemically heterogeneous rather than confined to a single product (Devillard et al., 2007). In preclinical models, microbial PUFA metabolism can shift host lipid composition and has been associated with resistance to diet-induced obesity and improved metabolic indices, with effects attributed in part to free fatty-acid receptor signaling; these data are murine and should be interpreted as mechanistic and preclinical rather than as evidence of a human metabolic outcome (Kishino et al., 2013; Miyamoto et al., 2019).

The clearest barrier-relevant example remains 10-HYA, which ameliorates epithelial barrier impairment partially through GPR40-MEK-ERK signaling *in vitro* and in experimental models (Miyamoto et al., 2015). This mechanism is specific to 10-HYA and should not be generalized to PUFA-derived mediators as a class. Other PUFA-derived intermediates have not been shown to act through the same pathway or to produce equivalent barrier effects.

A second, conceptually distinct group comprises oxylipins and endocannabinoid-like mediators. Many of these are host- or diet-derived rather than strict microbial products, so they are most accurately described as microbially modulated lipid networks whose abundance and activity are shaped by microbial ecology, dietary lipid quality, and host metabolism. In murine work, the endocannabinoid system links gut microbiota to adipogenesis, and the gut microbiota and endocannabinoidome respond rapidly and concomitantly to diet-induced obesity (Muccioli et al., 2010; Lacroix et al., 2019). Human fecal microbiota models indicate that microbial communities can contribute to the production of endocannabinoid-like mediators in a manner dependent on the dietary oil supplied and can process EPA/DHA-containing triglycerides into oxylipins with host-relevant activity; these are ex vivo or model-system data, not *in vivo* clinical demonstrations (Roussel et al., 2024; Roussel et al., 2025). In humans, dietary fatty-acid intake and selected gut bacterial families have been associated with circulating N-acyl-ethanolamines and 2-monoacyl-glycerols, and broader food patterns track with the gut microbiome-endocannabinoidome axis; both lines of evidence remain associative (Castonguay-Paradis et al., 2020; Castonguay-Paradis et al., 2023).

Taken together, PUFA-derived microbial lipid biology extends well beyond 10-HYA but is less clinically mature than SCFA and bile-acid biology. Human data remain largely associative or model-based, and a clear distinction between microbial production and microbial modulation of any given mediator is often unresolved. Establishing whether this diet-microbiome-host lipid network contributes to MASLD or other gut-liver phenotypes will require targeted lipidomics, compartmental sampling across luminal, mucosal, portal, and systemic matrices, and longitudinal designs rather than cross-sectional associations.

### 3.5 Choline-, carnitine-, and phospholipid-derived metabolites

Trimethylamine N-oxide (TMAO) occupies an intermediate position in this review. Structurally, TMAO is not a lipid in the narrow sense; it carries no acyl chain and does not belong to any standard lipid class. However, it is generated from lipid-associated or diet-related substrates -- principally phosphatidylcholine, choline, and L-carnitine -- making it inseparable from the broader choline and phospholipid metabolome (Wang et al., 2011; Koeth et al., 2013; Tang et al., 2013; Tang and Hazen, 2017). Its relevance to the gut-liver axis derives not from structure but from pathway: dietary substrate availability is shaped by precursor intake in the intestinal lumen; resident microbiota generate trimethylamine (TMA); TMA is absorbed and delivered to the liver through the portal circulation; and hepatic flavin-containing monooxygenase 3 (FMO3) oxidizes TMA to TMAO. This diet-microbe-liver co-metabolic sequence is therefore hepatoportal before it becomes systemic.

The hepatic step deserves emphasis because FMO3 is not simply a passive detoxification enzyme. Preclinical and translational data indicate that FMO3 is metabolically regulated, suppressed by insulin signaling, and altered in obesity and insulin-resistant states; in insulin-resistant mice, FMO3 knockdown suppressed hepatic FoxO1 activity and improved glycemic and lipid parameters (Miao et al., 2015). A complementary mouse study linked FMO3 and TMAO generation to hepatic cholesterol and triacylglycerol metabolism, inflammatory signaling, and endoplasmic reticulum stress (Warrier et al., 2015). These findings frame hepatic FMO3 as a metabolic node at which dietary substrate load, microbial TMA generation, insulin-sensitive hepatic metabolism, and inflammatory stress intersect, rather than merely as a quantitative converter of a gut-derived molecule.

Human translational evidence linking circulating TMAO to hepatic disease has also accumulated. In a cross-sectional study of obese patients with histologically assessed liver tissue, circulating TMAO and plasma choline were associated with NAFLD histologic features and NASH risk; the TMAO-NASH association was strongest among participants with type 2 diabetes, and secondary bile acids were associated with both TMAO levels and NASH features (León-Mimila et al., 2021). In the prospective PREVEND cohort, circulating TMAO was associated with all-cause mortality among participants with NAFLD, whereas this association was not observed in participants without NAFLD, suggesting a disease-context-dependent rather than universally harmful relationship (Flores‐Guerrero et al., 2021). A systematic review and meta-analysis found higher circulating TMAO in NAFLD patients than in controls, but also noted substantial between-study heterogeneity and possible publication bias (Theofilis et al., 2022). Taken together, these human data support a translational association between the TMA/TMAO pathway and hepatic metabolic disease, particularly in obesity, insulin resistance, and type 2 diabetes, without establishing a direct causal relationship.

Preclinical mechanistic data suggest one candidate pathway linking TMAO to hepatic dysfunction: bile-acid remodeling. In a dietary NAFLD mouse model, TMAO administration aggravated hepatic steatosis through altered bile-acid pool composition and inhibition of hepatic FXR signaling, indicating that the TMA/TMAO pathway may interact with the enterohepatic bile-acid axis at the receptor level (Tan et al., 2019). Because FXR contributes to hepatic fatty-acid oxidation, lipogenesis, bile-acid feedback, and inflammatory tone, impaired FXR signaling represents a plausible mechanism through which disrupted TMA/TMAO metabolism could contribute to hepatic lipid accumulation and inflammatory stress. These findings remain preclinical and should not be extrapolated directly to human MASLD without further translational validation.

Interpretation of circulating TMAO measurements remains complicated by confounders operating at different biological levels. Dietary precursor load, particularly from choline- and carnitine-rich foods, shapes TMA substrate availability, while fish and seafood may also contribute through preformed TMAO. Microbial ecology determines TMA-generating capacity independently of diet. Renal function strongly influences circulating TMAO through clearance, and hepatic FMO3 activity varies with insulin sensitivity, sex, and metabolic phenotype. Comorbid type 2 diabetes, cardiovascular disease, and chronic kidney disease may therefore influence both TMAO concentrations and clinical outcomes. These confounders make isolated TMAO measurements biologically informative but poorly specific. TMAO should therefore be regarded as a candidate mediator and biomarker within a broader gut-liver-cardiometabolic network, rather than interpreted as a direct index of microbial activity or a stand-alone vascular risk signal. Its specificity and causal contribution require dietary standardization, renal function adjustment, hepatic and metabolic phenotype stratification, and longitudinal validation. The relationship between TMAO and frailty in older adults with cardiovascular disease has been noted in observational data (He et al., 2020), but its mechanistic relevance to hepatic aging remains unresolved.

## 4 Epithelial barrier biology

### 4.1 Tight junctions, adherens junctions, and epithelial polarity

Tight junctions and adherens junctions are dynamic molecular gates rather than static seals. Their core constituents -- claudins, occludin, ZO-family scaffolding proteins, E-cadherin-associated complexes, and the actomyosin cytoskeleton -- define paracellular permeability, epithelial polarity, and responsiveness to cytokines and metabolic stress (Anderson and Van Itallie, 2009; Groschwitz and Hogan, 2009; Zihni et al., 2016) (**Table 2**). Within the claudin family, individual members differ markedly in function: barrier-forming claudins (e.g., claudin-1, claudin-3, claudin-4) restrict paracellular flux, whereas pore-forming claudins, most notably claudin-2, create cation- and water-permeable channels that increase paracellular conductance when upregulated. Claudin-2 expression is low in healthy colonic epithelium but is induced by inflammatory cytokines, and its upregulation represents a defined molecular mechanism of leak-pathway barrier dysfunction in inflammatory models (Anderson and Van Itallie, 2009; Zihni et al., 2016).

**Table 2:** Barrier and immune mechanisms through which microbial lipid metabolites may influence gut-liver immunometabolism.

Biological level	Mechanistic axis	Representative metabolites/receptors	Gut-liver relevance and interpretive caveat	References
Junctional barrier and epithelial polarity	Regulation of barrier-forming and pore-forming claudins, occludin, ZO proteins, adherens junctions, cytoskeletal tension, and paracellular permeability. Butyrate-related mechanisms include AMPK-dependent tight-junction assembly, HIF-1α-dependent regulation of tight-junction protein expression, IL-10R-dependent claudin-2 repression, and attenuation of cytokine-induced claudin-2 upregulation.	Butyrate; AMPK; HIF-1α; IL-10R; claudin-2; FXR; TGR5.	Best supported for butyrate-mediated epithelial effects in experimental models. Human compartment-specific validation remains limited; HIF-1α and claudin-2 pathways should be interpreted as context-dependent rather than uniformly protective.	(Anderson and Van Itallie, 2009; Groschwitz and Hogan, 2009; Peng et al., 2009; Cipriani et al., 2011; Gadaleta et al., 2011; Kelly et al., 2015; Zihni et al., 2016; Zheng et al., 2017; Yin et al., 2020; Huang et al., 2021; Song et al., 2025)
Mucus layer and antimicrobial defense	Control of mucin architecture, goblet-cell and Paneth-cell programs, defensins, antimicrobial peptides, epithelial glycosylation, and microbial spatial segregation. FXR-dependent antimicrobial defense is primarily ileal/small-intestinal; colonic bile-acid context differs.	SCFAs; ileal FXR-FGF19/FGF15 axis; colonic FXR/TGR5 context; Akkermansia-related products and extracellular vesicles; IL-22-linked epithelial programs.	Supports compartmentalization. FXR-dependent effects are segment-specific and depend on bile-acid composition, microbial transformation, epithelial compartment, mucus architecture, and inflammatory context. Single-taxon or single-product claims require caution.	(Inagaki et al., 2006; Bevins and Salzman, 2011; Adolph et al., 2013; Sayin et al., 2013; Reunanen et al., 2015; Johansson and Hansson, 2016; Ottman et al., 2017; Chelakkot et al., 2018; Gustafsson and Johansson, 2022)
Epithelial energy metabolism and stress resilience	Colonocyte oxidative metabolism, physiologic epithelial hypoxia, HIF stabilization, redox control, ER stress responses, autophagy, repair capacity, and oxygen-gradient control of microbial ecology.	Butyrate; SCFAs; HIF; PPAR-gamma; Nrf2; PXR-linked pathways.	Links microbial fermentation to epithelial resilience and luminal oxygen control; human tissue-level validation remains incomplete. Effects depend on epithelial metabolic state and inflammatory context.	(Kaser and Blumberg, 2009; Donohoe et al., 2011; Ploger et al., 2012; Kelly et al., 2015; Garg et al., 2016; Byndloss et al., 2017; Cuadrado et al., 2019; Singh et al., 2019)
Innate immune sensing and inflammasome tone	TLR and NOD-like receptor signaling, NLRP3/NLRP6 inflammasomes, NF-κB, MAPK, redox-sensitive inflammatory pathways, and cytokine generation under barrier-disruptive conditions.	SCFAs; bile acids; fatty-acid stress signals; LPS-context; TMAO-related systemic signals.	Can be homeostatic or maladaptive depending on barrier integrity, metabolic stress, duration of exposure, and compartment. Avoid treating PRR or inflammasome activation as uniformly pathological.	(Rakoff-Nahoum et al., 2004; Abreu, 2010; Elinav et al., 2011; Wen et al., 2011; Youm et al., 2013; Levy et al., 2015; Thaiss et al., 2016; Feng et al., 2018)
Adaptive, myeloid, and innate lymphoid immune-cell programming	Treg induction, Treg/Th17 balance, macrophage function, dendritic-cell tolerance, ILC-derived IL-22, epithelial repair, antimicrobial peptide responses, and epithelial glycosylation. Direct SCFA-IL-22 axis via GPR41/HDAC inhibition/AhR/HIF-1α pathway.	SCFAs; GPR41/FFAR3; HDAC inhibition; microbial bile-acid metabolites; bacterial sphingolipids; IL-22-linked epithelial pathways.	Strongest mechanistic support for SCFAs and bile-acid derivatives in Treg/Th17 and myeloid pathways. ILC effects are best interpreted mainly as indirect epithelial-myeloid-IL-22 circuits, with limited direct human validation.	(Rescigno et al., 2001; Coombes and Powrie, 2008; Maslowski et al., 2009; Sonnenberg et al., 2012; Arpaia et al., 2013; Furusawa et al., 2013; Smith et al., 2013; Bain and Mowat, 2014; Chang et al., 2014; Goto et al., 2014; Macia et al., 2015; Hang et al., 2019; Schulthess et al., 2019; Campbell et al., 2020; Song et al., 2020; Yang et al., 2020; Kim, 2021; Paik et al., 2022; Hegarty et al., 2023)
Portal delivery and hepatic immunometabolism	Loss of regulated compartmentalization, metabolic endotoxemia, bile-acid remodeling, microbial TMA production followed by hepatic FMO3-dependent oxidation, Kupffer-cell activation, stellate-cell crosstalk, and hepatic inflammatory priming.	Microbial products; SCFAs; bile acids; diet-derived lipid signals; TMA/TMAO-related co-metabolism; FMO3-linked hepatic oxidation.	Provides a translational bridge to MASLD and cardiometabolic disease. Causality requires longitudinal, intervention-based, and multi-compartment validation; peripheral blood and fecal signals are not equivalent to portal delivery or hepatic exposure.	(Cani et al., 2007; Cani et al., 2008; Le Roy et al., 2013; Mouzaki et al., 2013; Miao et al., 2015; Warrier et al., 2015; Boursier et al., 2016; Loomba et al., 2017; Tan et al., 2019; Albillos et al., 2020; De Munck et al., 2020; Flores‐Guerrero et al., 2021; León-Mimila et al., 2021; Theofilis et al., 2022; Tilg et al., 2022; Hsu and Schnabl, 2023; Pabst et al., 2023; Acierno et al., 2025c)

Butyrate is the short-chain fatty acid most directly studied in relation to junctional stability, and the best-characterized mechanism operates through AMP-activated protein kinase (AMPK). In Caco-2 epithelial monolayer experiments, butyrate activates AMPK and promotes tight-junction assembly, including redistribution of claudin-1, occludin, and ZO-1 to the apical junctional complex (Peng et al., 2009). AMPK-dependent tight-junction assembly is conceptually linked to epithelial energy sensing: colonocytes derive a substantial fraction of their ATP from butyrate-fueled beta-oxidation, and AMPK responds to energy availability by phosphorylating targets that regulate cytoskeletal organization and junction formation. This places butyrate at the intersection of microbial fermentation, colonocyte oxidative metabolism, and paracellular permeability control. The evidence for this pathway is derived primarily from *in vitro* epithelial models, and direct demonstration in intact human tissue remains limited (Peng et al., 2009).

A second, partially overlapping mechanism involves epithelial hypoxia-inducible factor (HIF) signaling. The intestinal epithelium exists in a state of physiologic hypoxia -- maintained by high oxygen consumption and the counter-current mucosal vasculature -- and HIF transcription factors support barrier-protective gene programs adapted to this environment. Microbiota-derived butyrate contributes to this physiologic epithelial hypoxia by sustaining colonocyte beta-oxidation, thereby limiting luminal oxygen availability and stabilizing epithelial HIF (Kelly et al., 2015). Among HIF isoforms, HIF-1α functions as a principal oxygen-sensitive transcription factor and, in epithelial models, participates in HIF-1α-dependent regulation of tight-junction protein expression in a butyrate-associated context (Yin et al., 2020). This provides a transcription-factor-linked arm through which microbial metabolism may influence junctional integrity, though this axis has been characterized primarily in preclinical systems and *in vitro* models. Stating that HIF-1α is unconditionally protective would overextend the evidence: HIF isoform identity, activation context, and downstream target repertoire all modulate the net outcome (Kelly et al., 2015).

A third mechanism connects butyrate to the repression of claudin-2. In experimental epithelial models, IL-10 receptor (IL-10R) signaling mediates butyrate-associated repression of claudin-2 expression, providing a specific molecular pathway through which butyrate may reduce claudin-2-mediated paracellular cation/water permeability (Zheng et al., 2017). Inflammatory cytokines -- including TNF-alpha, IFN-gamma, and IL-13 -- can drive cytokine-induced barrier dysfunction in epithelial models, in part by upregulating claudin-2, reducing transepithelial electrical resistance, and disrupting junctional protein localization (Huang et al., 2021). Butyrate has been shown to attenuate TNF-alpha/IFN-gamma-induced and IL-13-induced claudin-2 upregulation in epithelial monolayers, suggesting that it may partially counteract these barrier-disruptive cytokine effects under inflammatory conditions (Huang et al., 2021). These observations are derived from *in vitro* epithelial models, and their relevance to the human intestinal mucosa under inflammatory conditions, while biologically plausible, requires further translational investigation. Critically, butyrate does not block cytokine generation per se; rather, experimental evidence supports attenuation of specific cytokine-induced epithelial permeability responses in defined model systems.

Bile acids constitute a second class of luminal signals that modulate paracellular permeability, with effects dependent on pool composition, hydrophobicity, concentration, and receptor context. Farnesoid X receptor (FXR) activation has been associated with preserved barrier integrity in experimental colitis models (Gadaleta et al., 2011), whereas TGR5 signaling can modulate intestinal barrier and immune responses in a context-dependent fashion (Cipriani et al., 2011). The spatial heterogeneity of FXR expression and function across the ileum and colon, and its broader role in barrier-immune integration, are addressed in Sections 4.2 and 5.2 (Cipriani et al., 2011; Gadaleta et al., 2011; Song et al., 2025).

### 4.2 Mucus layer, antimicrobial peptides, and epithelial defense

The mucus layer reduces direct microbial contact with the epithelium, and Paneth-cell antimicrobial peptides help maintain spatial control of microbial communities. Goblet cells, mucins, Paneth cells, defensins, lysozyme, and epithelial immune mediators together create a chemically active barrier that supports tolerance while limiting pathobiont invasion (Bevins and Salzman, 2011; Adolph et al., 2013; Johansson and Hansson, 2016; Gustafsson and Johansson, 2022).

Microbial metabolites influence this compartment both directly and indirectly. SCFAs support epithelial homeostasis; FXR regulates antibacterial defense in the small intestine; and *Akkermansia muciniphila*-related products have been linked to epithelial adhesion, tight-junction regulation, and barrier modulation. These observations are biologically relevant, though caution is warranted before extending them into broad claims about any single taxon or metabolite (Inagaki et al., 2006; Reunanen et al., 2015; Ottman et al., 2017; Chelakkot et al., 2018).

The FXR component of this barrier program is spatially structured. In the distal small intestine, exposure to bile acids activates epithelial FXR-dependent programs linked to enteroprotection, antimicrobial peptide expression, and control of bacterial overgrowth. This ileal context differs from the colon, where bile acids have undergone more extensive microbial deconjugation and transformation and where secondary bile acids, dense microbial communities, mucus architecture, and inflammatory tone shape receptor-level effects. Therefore, FXR-mediated antimicrobial defense should not be interpreted as a uniform response along the entire intestinal tract; its biological output depends on bile-acid composition, epithelial segment, microbial density, and inflammatory context (Makishima et al., 1999; Parks et al., 1999; Kawamata et al., 2003; Inagaki et al., 2006; Ridlon et al., 2006; Thomas et al., 2009; Sayin et al., 2013; Li and Chiang, 2014; Wahlstrom et al., 2016; Chiang and Ferrell, 2020; Fiorucci et al., 2021; Collins et al., 2023).

### 4.3 Epithelial energy metabolism, HIF signaling, ER stress, and resilience

Epithelial barrier function is energy-dependent. Butyrate supports colonocyte oxidative metabolism and helps maintain a physiologically hypoxic epithelial microenvironment that favors anaerobic microbial ecology and HIF-mediated barrier protection. This creates a feedback circuit in which microbial fermentation sustains epithelial metabolism and, in turn, epithelial metabolism shapes luminal oxygenation and microbial composition (Donohoe et al., 2011; Ploger et al., 2012; Kelly et al., 2015; Byndloss et al., 2017).

Barrier failure can also emerge from converging cellular stress responses. ER stress, unfolded protein response dysregulation, oxidative stress, impaired autophagy, and inflammatory cytokines all weaken epithelial resilience. The available evidence supports an integrated model in which microbial metabolites modulate epithelial resilience in a context-dependent manner, rather than attributing this entire stress network to any single metabolite (Kaser and Blumberg, 2009; Garg et al., 2016; Cuadrado et al., 2019; Singh et al., 2019).

## 5 Immune signaling and metabolite-immune interfaces

### 5.1 Pattern-recognition receptors, NF-κB, Nrf2, and inflammasomes

Pattern-recognition receptors define the basal inflammatory tone of the intestinal mucosa. Toll-like receptor signaling and recognition of commensal microflora contribute to intestinal homeostasis, but excessive or persistent PRR stimulation can amplify inflammation when compartmentalization fails (Rakoff-Nahoum et al., 2004; Abreu, 2010; Thaiss et al., 2016).

Inflammasomes connect microbial sensing to metabolic stress. NLRP6 has been implicated in shaping the intestinal microenvironment through microbiota-modulated metabolites, whereas NLRP3 links fatty-acid-induced inflammation, insulin signaling interference, and low-grade inflammation in aging. SCFAs and other metabolites may attenuate inflammasome activation in some settings, but pro-inflammatory lipid environments can produce the opposite effect (Elinav et al., 2011; Wen et al., 2011; Youm et al., 2013; Levy et al., 2015; Feng et al., 2018).

### 5.2 GPCRs and nuclear receptors linking lipids, immunity, and metabolism

GPCRs and nuclear receptors form the receptor-level bridge between microbial lipid metabolism and host physiology. FFAR2/GPR43, FFAR3/GPR41, and GPR109A respond to SCFAs; FXR and TGR5 respond to bile acids; PXR, PPARs, and other lipid-sensing receptors integrate xenobiotic, microbial, and nutritional signals. Their effects depend on ligand concentration, compartment, cell type, and inflammatory context (Maslowski et al., 2009; Singh et al., 2014; Macia et al., 2015; Husted et al., 2017).

FXR and TGR5 are particularly relevant to the gut-liver axis because microbial bile-acid transformation changes receptor activation profiles across the intestine and liver. In the ileum, FXR is positioned within the enterohepatic feedback circuit: activation by luminal bile acids induces FGF19 in humans, or FGF15 in mice, thereby contributing to feedback repression of hepatic bile-acid synthesis and influencing hepatointestinal metabolic communication. In this compartment, FXR signaling is also linked to epithelial defense and antimicrobial programs. By contrast, the colon is exposed to a more microbially modified bile-acid pool, enriched in deconjugated and secondary bile acids, and receptor effects occur in a dense microbial and mucus-associated environment. Colonic FXR and TGR5 signaling may therefore influence epithelial barrier integrity, immune-cell activation, and inflammatory tone differently from ileal FXR-FGF19/FGF15 signaling (Makishima et al., 1999; Parks et al., 1999; Kawamata et al., 2003; Inagaki et al., 2006; Ridlon et al., 2006; Thomas et al., 2009; Cipriani et al., 2011; Gadaleta et al., 2011; Sayin et al., 2013; Li and Chiang, 2014; Wahlstrom et al., 2016; Chiang and Ferrell, 2020; Fiorucci et al., 2021; Collins et al., 2023).

This spatial heterogeneity helps explain why bile-acid receptor biology is difficult to translate therapeutically. Intestinal FXR activation can support antimicrobial defense and barrier integrity in selected experimental contexts, while other models suggest that intestinal FXR signaling may also contribute to metabolic dysfunction through pathways involving ceramide synthesis and hepatic lipogenesis. Conversely, intestine-selective FXR inhibition has improved obesity-related metabolic dysfunction and hepatic steatosis in preclinical models. These apparently divergent findings should not be treated as contradictions, but as evidence that FXR output depends on segmental exposure, ligand structure, receptor distribution, host metabolic state, and the microbial bile-acid transformation network (Venkatesh et al., 2014; Jiang et al., 2015a; Jiang et al., 2015b; Garg et al., 2016; Friedman et al., 2018).

### 5.3 Innate and adaptive immune-cell reprogramming

Microbial lipid-derived metabolites modulate both myeloid and lymphoid compartments. Macrophages are central to intestinal homeostasis and inflammation, and butyrate can regulate macrophage function through histone deacetylase inhibition and imprint antimicrobial programs under specific experimental conditions (Bain and Mowat, 2014; Chang et al., 2014; Schulthess et al., 2019; Hegarty et al., 2023). These effects are relevant to the gut-liver axis because metabolite-conditioned macrophages may influence epithelial repair, inflammatory tone, and the cytokine environment that governs downstream lymphoid responses.

Dendritic cells and T cells provide the adaptive arm of this metabolite-immune interface. Dendritic cells shape tolerance by sampling luminal and epithelial antigens, while SCFAs and bile-acid metabolites influence Treg differentiation, Treg/Th17 balance, and RORgamma+ regulatory T-cell homeostasis (Rescigno et al., 2001; Coombes and Powrie, 2008; Arpaia et al., 2013; Furusawa et al., 2013; Hang et al., 2019; Song et al., 2020). These pathways are among the more mature examples of microbial metabolite-dependent immune programming, although their translation to MASLD and cardiometabolic disease remains more inferential than directly interventional.

Innate lymphoid cells add a further barrier-homeostatic layer by integrating epithelial, microbial, and cytokine cues. Direct links between lipid-derived microbial metabolites and ILC biology are less well characterized than those involving Treg/Th17 pathways. However, ILCs are highly relevant to this review because they regulate epithelial programs that are themselves metabolite-sensitive. ILC-derived IL-22 supports anatomical containment of commensals, epithelial repair, antimicrobial peptide responses, and epithelial glycosylation, thereby reinforcing the mucus-associated and antimicrobial defense mechanisms discussed in Section 4.2 (Inagaki et al., 2006; Bevins and Salzman, 2011; Sonnenberg et al., 2012; Adolph et al., 2013; Goto et al., 2014; Reunanen et al., 2015; Johansson and Hansson, 2016; Ottman et al., 2017; Chelakkot et al., 2018; Gustafsson and Johansson, 2022). In this sense, microbial lipid metabolites may influence ILC-dependent barrier function indirectly by altering epithelial energy metabolism, mucus integrity, bile-acid receptor signaling, macrophage and dendritic-cell cytokine output, and the availability of IL-22-inducing signals.

Recent experimental evidence also suggests a more direct SCFA-IL-22 axis. Microbiota-derived SCFAs have been shown to promote IL-22 production by CD4+ T cells and ILCs through GPR41-dependent signaling and HDAC inhibition, with involvement of AhR and HIF-1α-dependent transcriptional programs (Yang et al., 2020). This finding provides a mechanistic bridge between microbial fermentation, immune-cell cytokine output, and epithelial defense. Nevertheless, the evidence remains largely preclinical and immune-cell-model based; it should not be interpreted as proof that lipid-derived microbial metabolites directly reprogram ILCs in human MASLD or frailty. A cautious interpretation is that ILCs represent an indirect barrier-amplifying node through which metabolite-regulated epithelial and myeloid signals may shape mucosal containment and portal inflammatory exposure.

## 6 Barrier dysfunction, microbial lipid metabolites, and hepatic immunometabolism

At the gut-liver interface, barrier failure is better understood as loss of regulated compartmentalization than as simple “leakiness.” Increased permeability, mucus dysfunction, dysbiosis, altered bile-acid pools, and lipotoxicity can form a recursive loop: the liver shapes bile, bile shapes microbial ecology, microbes transform bile acids and dietary lipids, and the barrier regulates portal delivery of metabolites and microbial patterns (Albillos et al., 2020; Tilg et al., 2022; Pabst et al., 2023).

This recursive loop is clinically important because it implies that intestinal and hepatic abnormalities may be mutually reinforcing. A dysbiotic microbiota may alter bile-acid transformation and barrier signaling; altered hepatic bile secretion may reshape microbial ecology; and low-grade portal inflammation may impair hepatic insulin signaling and lipid handling.

### 6.1 Increased intestinal permeability and portal delivery of microbial signals

Metabolic endotoxemia is one influential model linking diet, permeability, microbiota, and systemic metabolic inflammation. High-fat diet models connect gut microbiota changes with low-grade inflammatory signaling and insulin resistance, but translation to humans requires caution given the complexity of LPS measurement, diet confounding, adiposity, and comorbid metabolic disease (Cani et al., 2007; Cani et al., 2008; Camilleri, 2019).

In MASLD, evidence supports an association between altered permeability and liver disease severity, though the causal direction remains unresolved. Barrier dysfunction may contribute to hepatic inflammation, yet steatosis, bile-acid alterations, systemic inflammation, and metabolic disease can also worsen intestinal integrity. This bidirectionality is central to the gut-liver framework (Aron-Wisnewsky et al., 2020; De Munck et al., 2020; Tilg et al., 2022).

### 6.2 MASLD and insulin resistance as immunometabolic models

MASLD is not merely hepatic fat accumulation: it is an immunometabolic syndrome in which insulin resistance, adipose-tissue dysfunction, lipotoxicity, oxidative stress, microbial signals, bile-acid remodeling, and low-grade inflammation converge. Human studies link NAFLD/MASLD severity to dysbiosis, metagenomic signatures, metabolomic changes, and microbial functional shifts, while experimental models suggest that the microbiota can contribute to steatosis development (Le Roy et al., 2013; Mouzaki et al., 2013; Boursier et al., 2016; Loomba et al., 2017; Acierno et al., 2025c).

The transition from NAFLD to MASLD strengthens this interpretive frame by explicitly foregrounding metabolic dysfunction, cardiometabolic risk, and inflammatory comorbidity rather than treating steatosis as an isolated hepatic phenotype (Acierno et al., 2025c). Insulin resistance is also modulated by lipid-sensitive inflammatory pathways. Saturated fatty-acid-induced ceramide biosynthesis, TLR4-associated inflammation, and NLRP3 activation can interfere with insulin signaling, whereas SCFAs and bile-acid receptor signaling may support metabolic homeostasis in selected contexts (Peng et al., 2009; Thomas et al., 2009; Holland et al., 2011; Wen et al., 2011; Acierno et al., 2025a).

### 6.3 Bile-acid signaling and hepatic lipid metabolism

Bile-acid signaling is the strongest mechanistic connection between microbial lipid metabolism and hepatic metabolic regulation. Through FXR and TGR5, bile acids regulate bile-acid synthesis, lipid flux, glucose metabolism, energy expenditure, immune tone, and hepatointestinal feedback. Microbial transformation of bile acids can therefore reshape both intestinal barrier signaling and hepatic lipid metabolism simultaneously (Li and Chiang, 2014; Wahlstrom et al., 2016; Chiang and Ferrell, 2020; Fiorucci et al., 2021).

Therapeutically, this axis is attractive but complex. Obeticholic acid and related bile-acid receptor-targeted strategies demonstrate the translational relevance of FXR modulation, but also highlight the need to balance efficacy, safety, tolerability, microbiome effects, and patient selection (Schaap et al., 2014; Friedman et al., 2018; Younossi et al., 2019).

## 7 Cardiometabolic disease, aging, skeletal muscle, and frailty

### 7.1 Obesity, type 2 diabetes, and vascular inflammation

Obesity and type 2 diabetes provide a systemic context in which diet, microbiota, barrier function, and lipid metabolites converge. The gut microbiota has been experimentally linked to fat storage and metabolic inflammation, while donor fecal microbiota transfer has demonstrated insulin-sensitivity effects that depend on baseline microbial composition (Backhed et al., 2004; Cani et al., 2007; Vrieze et al., 2012; Kootte et al., 2017).

Type 2 diabetes and steatotic liver disease share insulin resistance, adipose inflammation, altered hepatic lipid flux, mitochondrial stress, and vascular risk. This overlap supports the view that microbial lipid signaling should be evaluated within a cardiometabolic continuum rather than in liver-specific silos (Acierno et al., 2020).

### 7.2 Aging, inflammaging, immunosenescence, skeletal muscle, and frailty

Inflammaging describes the chronic low-grade immune-metabolic activation that underlies age-related disease, and hallmarks-of-aging frameworks connect mitochondrial dysfunction, cellular senescence, impaired proteostasis, and altered intercellular communication to clinical vulnerability (Franceschi et al., 2000; Lopez-Otin et al., 2013; Franceschi et al., 2018; Lopez-Otin et al., 2023). Aging is also associated with changes in microbiota composition and function, intestinal permeability, mucus barrier integrity, systemic inflammation, and macrophage dysfunction. Experimental transfer studies and human observational data support a plausible gut-barrier-inflammaging axis, although human causality remains difficult to establish (Biagi et al., 2010; O'Toole and Jeffery, 2015; Fransen et al., 2017; Thevaranjan et al., 2017; Sovran et al., 2019; Untersmayr et al., 2022).

Skeletal muscle is a relevant downstream compartment in this axis because it integrates insulin-mediated glucose disposal, mitochondrial substrate oxidation, amino-acid turnover, protein synthesis, proteostasis, and myokine signaling. Sarcopenia and physical frailty arise from converging processes that include anabolic resistance, impaired insulin/IGF-1-Akt-mTOR signaling, mitochondrial dysfunction, myosteatosis, chronic cytokine exposure, reduced physical activity, malnutrition, and increased proteolytic pressure through ubiquitin-proteasome and autophagy-lysosome pathways (Fielding et al., 2011; Cruz-Jentoft et al., 2019; Piggott and Tuddenham, 2020; Strasser and Ticinesi, 2023). Microbial lipid-derived metabolites should not be presented as primary causes of sarcopenia, but they may modulate several upstream regulators of muscle quality, particularly systemic inflammatory tone, insulin sensitivity, energy substrate handling, and gut-derived immune activation.

The most biologically plausible microbial link involves SCFAs. In preclinical models, depletion or absence of gut microbiota impairs skeletal muscle mass and function, while microbiota transfer or SCFA exposure can partially restore muscle-related metabolic and functional phenotypes (Lahiri et al., 2019). More recent experimental work suggests that SCFAs may attenuate age-related muscle loss and dysfunction through protein-synthesis-related mTOR signaling in sarcopenic mice and myotube models (Liu et al., 2024). Human evidence is less definitive but translationally relevant: community-based multi-omics data have linked gut microbial butyrate-producing capacity to skeletal muscle mass in healthy menopausal women (Lv et al., 2021). These findings support a gut-muscle axis, but they do not prove that increasing SCFAs is sufficient to reverse sarcopenia in older adults or in MASLD.

The gut-liver context is particularly important when sarcopenia coexists with metabolic liver disease or advanced chronic liver disease. In this setting, reduced muscle quality may reflect not only aging, but also insulin resistance, chronic inflammation, altered amino-acid availability, ammonia handling, bile-acid remodeling, mitochondrial stress, and reduced physical reserve. In patients with liver cirrhosis, sarcopenia has been associated with altered gut microbiome composition, bile-acid profiles, and metabolomic signatures, suggesting a functional gut-microbiome-bile-acid-muscle interaction, although causality and directionality remain unresolved (Aliwa et al., 2023). For MASLD, the same framework is plausible but less directly demonstrated: obesity, diabetes, steatotic liver disease, and sarcopenic obesity may converge through insulin resistance, adipose inflammation, hepatic lipid stress, and barrier-related inflammatory signaling rather than through a single microbial metabolite.

The clinical relevance is strongest when aging biology is linked to functional endpoints such as frailty, sarcopenia, cardiovascular disease, and metabolic dysfunction. The NU-AGE intervention connects Mediterranean diet, microbiome remodeling, frailty reduction, and health status in older adults, while TMAO has been associated with frailty in older adults with cardiovascular disease -- supporting integrated biomarker panels rather than single-metabolite risk models (Fielding et al., 2011; Cruz-Jentoft et al., 2019; Ghosh et al., 2020; He et al., 2020; Piggott and Tuddenham, 2020; Strasser and Ticinesi, 2023). Age-related microbiome research also suggests that diet, residential context, medication exposure, physical activity, protein intake, and functional status may be as important as chronological age. Preclinical studies add mechanistic plausibility, but they do not yet define an intervention-ready microbial lipid signature for frail or sarcopenic older patients (Claesson et al., 2012; Zeng et al., 2019; Badal et al., 2020; Bosco and Noti, 2021; Chittimalli et al., 2023; Acierno et al., 2025b; Sommer et al., 2025).

## 8 Therapeutic and translational perspectives

The translational question is whether the microbial lipid metabolome can be modulated to improve barrier function, immune tone, and metabolic outcomes. Current strategies include diet, fiber, prebiotics, probiotics, synbiotics, postbiotics, bile-acid receptor modulation, fecal microbiota transplantation, next-generation microbial therapeutics, and metabolomic biomarker development. **Table 3** organizes these strategies by mechanistic rationale, potential utility, current limitations, and supporting references.

**Table 3:** Translational maturity of candidate interventions, biomarker strategies, and methodological platforms.

Strategy	Mechanistic rationale	Potential utility	Current limitation	References
Dietary fiber and dietary lipid quality	Modulates fermentable substrate availability, SCFA generation, bile-acid pools, PUFA-derived mediators, endocannabinoidome-related tone, protein fermentation, and inflammatory lipid signaling.	Low-risk upstream intervention for prevention and early metabolic modulation; relevant to MASLD, cardiometabolic risk, frailty, and sarcopenic vulnerability when combined with protein adequacy and physical-function assessment.	Response heterogeneity; adherence; caloric balance; protein intake; comorbidity; medication exposure; baseline microbiome function.	(Makki et al., 2018; Ghosh et al., 2020; Piggott and Tuddenham, 2020; Fu et al., 2021; Castonguay-Paradis et al., 2023; Strasser and Ticinesi, 2023; Mann et al., 2024)
Prebiotics, probiotics, synbiotics, postbiotics	Targets microbial ecology, substrate use, strain-level functions, and microbial structural/metabolic products.	Potential adjunctive strategy in MASLD and cardiometabolic disease research.	Heterogeneous strains, formulations, endpoints, and patient selection; not yet a precise microbial-lipid therapy.	(Ma, 2013; Hill et al., 2014; Gibson et al., 2017; Liu et al., 2019; Sharpton et al., 2019; Swanson et al., 2020; Salminen et al., 2021)
FMT and next-generation microbial therapeutics	Transfers or engineers microbial functions that may affect insulin sensitivity, SCFA production, bile-acid conversion, barrier biology, or inflammatory tone.	Useful investigational platform for causal and functional testing.	Safety, donor/recipient dependence, regulatory constraints, limited metabolic indications, and uncertain durability of effect.	(Vrieze et al., 2012; Cammarota et al., 2017; Kootte et al., 2017; Suez et al., 2018)
Bile-acid receptor modulation (FXR, TGR5)	Targets FXR/TGR5 and hepatointestinal feedback mechanisms linking bile acids, barrier function, antimicrobial defense, glucose metabolism, and lipid handling; ileal vs. colonic FXR context is distinct.	Mechanistically strong therapeutic entry point, especially when bile-acid pool composition and intestinal versus hepatic receptor activation are considered.	Segment-specific and ligand-specific receptor effects; safety/tolerability issues; pruritus/lipid effects for some agents; need for careful patient selection.	(Sayin et al., 2013; Schaap et al., 2014; Friedman et al., 2018; Younossi et al., 2019; Li and Chiang, 2024)
Composite metabolomic/lipidomic biomarkers	Integrates microbial function, targeted lipid mediator profiling, bile-acid pools, SCFAs/BCFAs, sphingolipids/ceramides, PUFA-derived mediators, TMA/TMAO-related co-metabolites, barrier markers, immune phenotyping, and clinical endpoints.	Potential future stratification tool for MASLD, cardiometabolic risk, frailty, and sarcopenic phenotypes when interpreted with diet, medication exposure, renal function, liver stage, and functional outcomes.	Requires longitudinal validation, analytical standardization, reference materials, isotope-labelled internal standards, batch correction, multi-compartment interpretation, and validation against clinical and functional endpoints.	(Puri et al., 2007; Sumner et al., 2007; Puri et al., 2009; Kalhan et al., 2011; Raman et al., 2013; Köfeler et al., 2021; Mirzayi et al., 2021; Lippa et al., 2022)
Multi-compartment profiling and flux-based studies	Pairs stool, plasma/serum, and, when feasible, mucosal, bile, or tissue sampling with stable-isotope tracing to separate production, absorption, utilization, hepatic extraction, and systemic availability.	Clarifies whether microbial lipid signals are biomarkers, adaptive responses, or plausible mediators of gut-liver communication; particularly relevant for SCFAs, TMA/TMAO, bile-acid turnover, and PUFA-derived products.	Portal venous sampling is rarely feasible; isotope studies are technically demanding; tissue sampling is opportunistic and often restricted to clinically indicated procedures.	(Boets et al., 2017; Sakata, 2019)
Organoids, co-cultures, and gut-liver microphysiological platforms	Reconstructs epithelial, mucus, immune, microbial, oxygen-gradient, and hepatointestinal interactions more realistically than conventional monolayers.	Useful bridge between reductionist in vitro studies and human cohorts; can test barrier function, bile-acid receptor signaling, microbial metabolism, cytokine output, and epithelial injury readouts.	Platform complexity; limited standardization; incomplete immune, vascular, neural, and microbial representation; uncertain scalability for clinical prediction.	(Jalili-Firoozinezhad et al., 2019; Trapecar et al., 2021; Fofanova et al., 2024)

A practical framework distinguishes low-risk upstream interventions from precision pharmacology. Diet and lifestyle act broadly and suit prevention, but produce heterogeneous metabolomic responses. Microbiome products and FMT require stricter indication, strain or donor characterization, safety monitoring, and endpoint selection. Pharmacological bile-acid modulation offers stronger mechanistic targeting but carries greater risk of off-target metabolic consequences.

### 8.1 Diet, fiber, and lipid quality

Diet is the most physiological upstream modulator of microbial lipid metabolism. Fermentable fibers drive SCFA production, while dietary lipid quality may alter bile-acid pools, PUFA-derived microbial products, endocannabinoidome tone, and inflammatory signaling. Diet is best understood as an ecosystem-level intervention rather than a single-nutrient exposure (Makki et al., 2018; Fu et al., 2021; Castonguay-Paradis et al., 2023; Mann et al., 2024).

In MASLD and cardiometabolic disease, dietary interventions should account for caloric balance, fiber quality, saturated versus unsaturated fat, protein adequacy, insulin resistance, medications, and baseline microbiome function. In older adults, the same principles must be balanced against malnutrition, sarcopenia, frailty, dysphagia, and polypharmacy (Ghosh et al., 2020; Piggott and Tuddenham, 2020; Strasser and Ticinesi, 2023).

### 8.2 Prebiotics, probiotics, synbiotics, postbiotics, and FMT

Terminology matters. ISAPP consensus definitions distinguish probiotics as live microorganisms that confer benefit when administered adequately, prebiotics as selectively utilized substrates, synbiotics as combined microbial-substrate strategies, and postbiotics as preparations of inanimate microorganisms or their components that confer a health benefit. Precise use of these terms avoids promotional imprecision (Hill et al., 2014; Gibson et al., 2017; Swanson et al., 2020; Salminen et al., 2021).

In MASLD, meta-analyses of probiotics, synbiotics, and gut microbiome-targeted therapies suggest potential metabolic and hepatic effects, but heterogeneity in strains, formulations, duration, endpoints, and patient selection limits generalizability. These interventions are best treated as promising research tools, not established microbial-lipid therapies (Ma, 2013; Liu et al., 2019; Sharpton et al., 2019).

FMT is more radical and should be approached cautiously outside established indications. Evidence in metabolic syndrome suggests potential effects on insulin sensitivity, but response depends on donor and recipient microbial features. Consensus documents support FMT in defined clinical contexts, while metabolic or aging applications remain investigational (Vrieze et al., 2012; Cammarota et al., 2017; Kootte et al., 2017; Suez et al., 2018).

### 8.3 Bile-acid-targeted interventions and metabolomic biomarkers

Bile-acid receptor modulation has a strong mechanistic rationale because FXR and TGR5 sit at the intersection of bile flow, microbiota, epithelial defense, glucose metabolism, lipid metabolism, and inflammation. However, the same receptor system can produce different effects depending on intestinal versus hepatic activation, bile-acid pool composition, disease stage, and comedication (Schaap et al., 2014; Friedman et al., 2018; Younossi et al., 2019; Li and Chiang, 2024).

Metabolomic and lipidomic biomarkers are attractive but not yet ready for routine clinical decision-making. Plasma and fecal metabolomic studies in NAFLD/NASH demonstrate feasibility, but clinical use requires reproducibility, compartment-specific interpretation, validation against outcomes, and integration with diet, medications, renal function, hepatic phenotype, permeability markers, and immune profiling (Puri et al., 2007; Puri et al., 2009; Kalhan et al., 2011; Raman et al., 2013).

The most promising biomarker strategy is composite: microbial taxonomy or metagenomics, targeted lipid metabolomics, bile-acid pool composition, SCFAs, TMAO or related co-metabolites, epithelial barrier markers, inflammatory mediators, and organ-specific endpoints. Such panels should be evaluated longitudinally to determine whether they predict progression, treatment response, or resilience.

## 9 Methodological challenges and future directions

**Figure 2:**
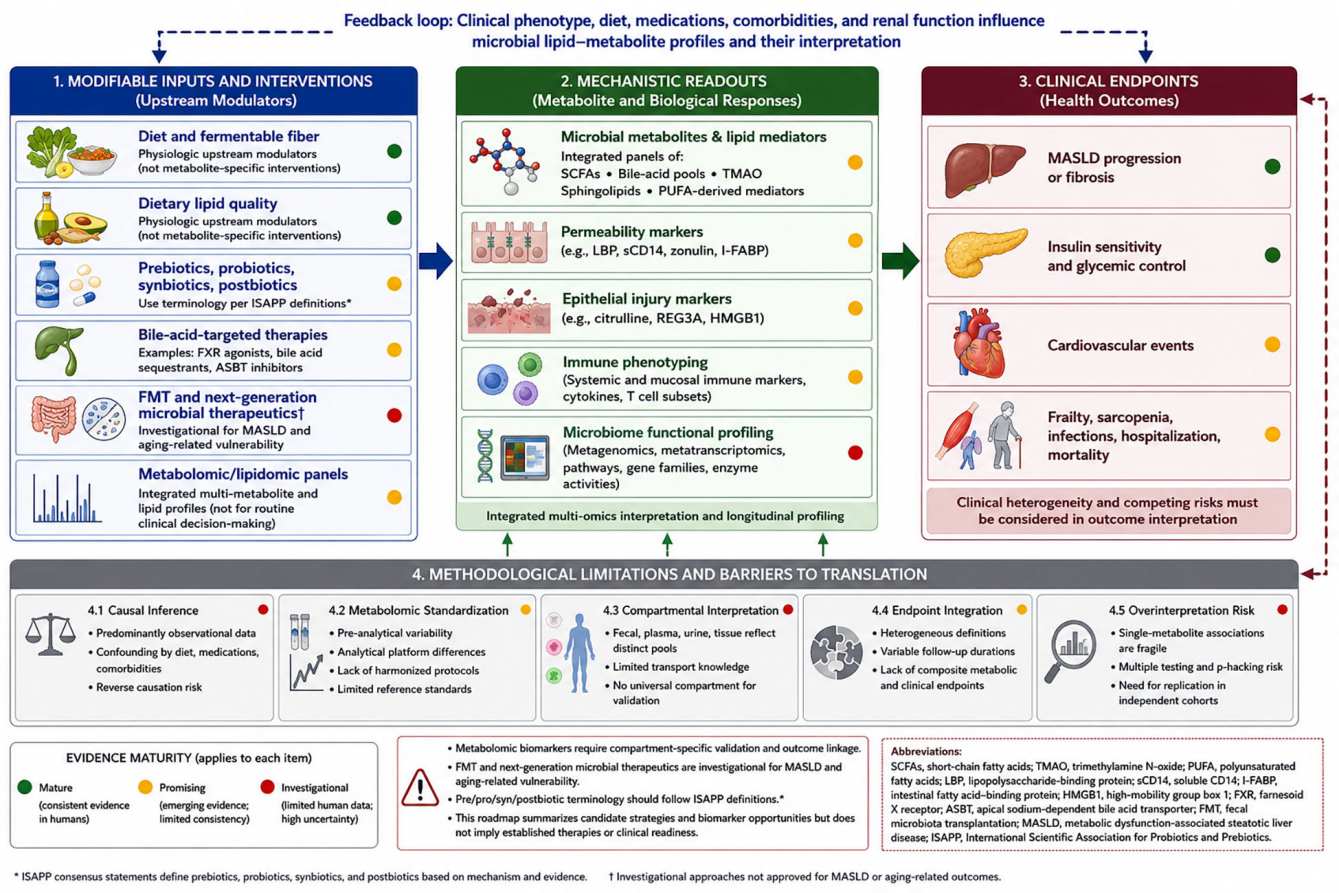
Translational roadmap from microbial lipid signatures to clinical phenotyping. Proposed multi-compartment workflow integrating stool, peripheral blood, and, when clinically feasible, mucosal, bile, or tissue sampling with targeted metabolomics/lipidomics, bile-acid profiling, SCFA and TMA/TMAO quantification, sphingolipid/ceramide and PUFA-derived mediator analysis, permeability and epithelial-injury markers, immune phenotyping, and MASLD, cardiometabolic, frailty, sarcopenia, and functional endpoints. The roadmap emphasizes longitudinal validation, standardized preanalytics, reference materials, isotope-labelled internal standards, metabolite-identification confidence, batch correction, and adjustment for diet, medications, renal function, liver phenotype, physical activity, nutritional status, and comorbidity burden before microbial lipid signatures can be used for clinical stratification.

The main methodological limitation in this field is that most studies infer gut-liver signaling from measurements obtained in a single compartment. This is particularly problematic for microbial lipid-derived or lipid-related metabolites because luminal abundance, mucosal exposure, epithelial utilization, portal delivery, hepatic extraction, and systemic availability are biologically distinct layers (**Figure 2**). A fecal metabolite concentration may reflect production, residual excretion, impaired absorption, altered transit, diet, microbial ecology, or analytical handling rather than direct receptor engagement at the epithelial or hepatic level. This limitation is especially relevant for SCFAs and BCFAs, where fecal output should not be interpreted as a proxy for production rate, colonocyte utilization, portal flux, or systemic signaling. Stable-isotope studies have shown that colonically derived SCFAs may undergo substantial absorption and metabolism before reaching the systemic circulation, supporting the need to distinguish fecal excretion from metabolic availability (Boets et al., 2017; Sakata, 2019).

Future studies should therefore adopt multi-compartment designs whenever feasible. At minimum, paired stool and peripheral blood sampling should be combined with standardized dietary assessment, medication recording, renal function, liver phenotype, metabolic status, and inflammatory markers. In selected clinical or procedural contexts, mucosal biopsies, bile samples, portal or hepatic venous samples, liver tissue, or intestinal aspirates may provide higher-resolution compartmental information, although these matrices are usually opportunistic and cannot be generalized to population-level studies. For gut-liver inference, the key question is not whether a metabolite is detectable in stool, but whether its production, transformation, epithelial handling, hepatic processing, or systemic persistence maps onto a plausible biological mechanism and a clinically meaningful phenotype.

Analytically, this field requires more targeted and standardized metabolomic and lipidomic workflows. Broad untargeted profiling remains useful for discovery, but mechanistic interpretation requires validated assays for SCFAs and BCFAs, bile-acid species, TMA/TMAO and related choline/carnitine metabolites, bacterial sphingolipids and host ceramides, hydroxy-fatty acids, oxylipins, and endocannabinoid-like mediators. Preanalytical variables -- fasting status, recent diet, stool collection method, storage temperature, freeze-thaw cycles, extraction protocol, derivatization procedure, instrument platform, batch structure, and normalization strategy -- can all influence measured concentrations. Future work should therefore include isotope-labelled internal standards, pooled quality-control samples, reference materials where available, transparent batch-correction procedures, metabolite-identification confidence levels, and explicit reporting of sample handling and analytical parameters (Sumner et al., 2007; Köfeler et al., 2021; Mirzayi et al., 2021; Lippa et al., 2022).

Longitudinal and perturbation-based designs are also needed. Cross-sectional associations between microbial taxa, metabolites, permeability markers, and MASLD or cardiometabolic outcomes cannot establish directionality. Diet, weight change, insulin resistance, alcohol intake, proton-pump inhibitors, metformin, GLP-1 receptor agonists, antibiotics, statins, bile-acid-modifying drugs, renal function, age, sex, physical activity, and frailty all influence microbial and lipid-metabolic readouts. Controlled dietary interventions, fiber or prebiotic challenges, pharmacological perturbation studies, bariatric-surgery cohorts, and longitudinal MASLD cohorts with repeated metabolomic profiling could help distinguish causal mediators from adaptive responses or disease biomarkers. These designs should integrate liver-related endpoints, including steatosis, inflammation, fibrosis, bile-acid pool composition, and hepatocellular injury markers, with intestinal permeability, epithelial injury, immune phenotyping, and systemic metabolic outcomes.

Stable-isotope and flux-based approaches represent a particularly important next step. Isotope-labelled substrates can help separate microbial production from host absorption, interconversion, oxidation, incorporation into lipid pools, and systemic clearance. For SCFAs, this approach can clarify the relationship between fermentation, colonocyte utilization, and systemic availability. For TMA/TMAO, isotope-labelled choline, carnitine, or TMA precursors could help distinguish dietary substrate load, microbial TMA-generating capacity, hepatic FMO3-dependent oxidation, renal clearance, and disease-context effects. For PUFA-derived mediators and bacterial sphingolipids, isotope-resolved lipidomics may help determine whether specific molecules are directly microbially produced, microbially transformed, or indirectly modulated through host-diet lipid networks. Such designs are technically demanding, but they are more informative than isolated concentration measurements.

Experimental platforms should also evolve beyond reductionist monolayer systems. Intestinal organoids, epithelial-immune co-cultures, anaerobic or physoxic host-microbe co-culture systems, gut-on-chip platforms, and linked gut-liver microphysiological systems can model aspects of epithelial differentiation, mucus biology, oxygen gradients, microbial metabolism, bile-acid signaling, cytokine responses, and hepatic processing that are not captured by conventional cell culture. These systems are not substitutes for human cohorts, and their technical complexity, incomplete immune and vascular representation, limited standardization, and uncertain scalability remain major limitations. Nevertheless, they offer an intermediate translational layer for testing whether candidate microbial lipid signals can modify epithelial barrier function, antimicrobial programs, bile-acid receptor signaling, hepatic inflammatory responses, or metabolite flux under controlled conditions (Jalili-Firoozinezhad et al., 2019; Trapecar et al., 2021; Fofanova et al., 2024).

A further priority is clinical endpoint selection. Studies of microbial lipid metabolism should not rely exclusively on taxonomic dysbiosis or single-metabolite abundance. For MASLD, endpoints should include liver fat, inflammatory activity, fibrosis stage, non-invasive fibrosis markers, insulin resistance, adipose-tissue dysfunction, bile-acid profiles, and cardiometabolic risk. For aging and frailty, outcomes should include muscle mass, muscle strength, gait speed, physical performance, nutritional status, inflammatory tone, medication burden, and functional decline. This is particularly important for sarcopenia and frailty, where microbial lipid-derived metabolites are more plausibly modulators of upstream inflammatory and metabolic networks than primary disease drivers.

Finally, future work should avoid single-metabolite causal narratives. SCFAs, bile acids, bacterial sphingolipids, PUFA-derived mediators, and TMA/TMAO-related co-metabolites should be interpreted as interacting components of a compartmentalized gut-liver immunometabolic network. Their biological effects depend on diet, microbial enzymatic capacity, anatomical site, epithelial energy state, mucus and junctional integrity, receptor distribution, hepatic extraction, renal clearance, inflammatory context, and host metabolic phenotype. The most useful translational models will therefore integrate microbial function, targeted lipidomics, barrier biology, immune profiling, organ-specific endpoints, and longitudinal clinical outcomes. This approach is more complex than fecal biomarker interpretation, but it is necessary if microbial lipid metabolites are to move from associative signals toward mechanistically interpretable and clinically relevant gut-liver phenotypes.

## 10 Conclusions

Lipid-derived and lipid-related microbial metabolites provide a coherent framework for interpreting how intestinal ecology communicates with barrier integrity, mucosal immunity, hepatic metabolism, and systemic cardiometabolic or aging-associated vulnerability. Classical SCFAs and bile-acid signaling remain the strongest mechanistic anchors of this framework, particularly for epithelial energy metabolism, junctional regulation, immune-cell programming, antimicrobial defense, and hepatointestinal receptor signaling. However, the revised model also requires a broader and more differentiated view: branched-chain SCFAs should be interpreted as protein- and BCAA-fermentation signals rather than simple equivalents of butyrate; bacterial sphingolipids and PUFA-derived mediators are biologically compelling but translationally less mature; and TMA/TMAO illustrates gut-liver co-metabolism through microbial TMA generation and hepatic oxidation rather than a direct epithelial-barrier pathway.

The most defensible central thesis is not that any single metabolite drives MASLD, insulin resistance, frailty, sarcopenia, or cardiometabolic disease. Rather, altered microbial lipid metabolism and impaired intestinal compartmentalization may shift the host from regulated immunometabolic adaptation toward chronic low-grade inflammatory stress. This shift is likely to be context-dependent, shaped by diet, microbial enzymatic capacity, bile-acid pool composition, epithelial energy state, mucus and junctional integrity, renal function, hepatic phenotype, medication exposure, aging biology, and physical-functional reserve.

The field is ready for integrated mechanistic and translational study, but not for clinical algorithms based on isolated metabolite measurements. Fecal abundance, mucosal exposure, portal delivery, hepatic extraction, and systemic availability represent different biological layers and should not be treated as interchangeable. Future work should therefore prioritize longitudinal cohorts, controlled dietary or pharmacological perturbation studies, multi-compartment sampling, stable-isotope tracing, targeted metabolomics and lipidomics, standardized preanalytical workflows, and organoid or microphysiological platforms capable of modelling epithelial-immune-microbial and gut-liver interactions.

A mature translational model will require moving from taxonomic dysbiosis and single-biomarker interpretation toward compartment-aware, function-oriented, patient-stratified immunometabolic phenotyping. Such an approach is more demanding, but also more clinically realistic: it preserves mechanistic precision while accommodating the biological heterogeneity of patients with MASLD, cardiometabolic disease, aging-associated frailty, and sarcopenic vulnerability.
